# Evaluation of Nonthermal Technologies to Reduce or Replace Nitrite in Meat Products

**DOI:** 10.17113/ftb.63.01.25.8744

**Published:** 2025-03

**Authors:** Bruna Fernandes Andrade, Lorrany Ramos do Carmo, Marcelo Stefani Tanaka, Robledo de Almeida Torres Filho, Alcinéia de Lemos Souza Ramos, Eduardo Mendes Ramos

**Affiliations:** 1 Department of Food Science, School of Agricultural Sciences of Lavras, Federal University of Lavras, 35690-900, Lavras, Minas Gerais, Brazil; 2 Institute of Exact and Technological Sciences, Federal University of Viçosa, Forestry Campus, 35690-000, Florestal, Minas Gerais, Brazil

**Keywords:** nitrosamines, *Clostridium botulinum*, irradiation, plasma, high pressure

## Abstract

Nitrite and nitrate salts are preservatives that act as antimicrobial (bacteriostatic and bactericidal activity) and antioxidant agents in the processing of meat products and confer sensory properties to meat (by creating and preserving colours and flavours). Nitrite is mainly used as a preservative to prevent the growth of *Clostridium botulinum* and the production of its toxins. However, nitrite and nitrate are also associated with the production of N-nitroso compounds, such as carcinogenic N-nitrosamines, which can have adverse health effects. Therefore, the health risks of these preservatives must be weighed against the need to prevent foodborne pathogens, especially spores of *C. botulinum*, from infecting food. In this review, we discuss the advantages and disadvantages of using nonthermal technologies as a strategy to partially or totally replace nitrite in meat products, particularly regarding antimicrobial efficacy and N-nitrosamine formation. Methods such as high-pressure processing, pulsed electric fields and cold plasma have been studied for these purposes, but these technologies can alter the sensory properties and stability of foods. Nevertheless, irradiation at lower doses has great potential as a tool for reformulation of cured meat products. It contributes to the reduction of the residual nitrite and consequently to the production of N-nitrosamines while ensuring microbiological safety without significant changes in the product quality.

## INTRODUCTION

Curing salts, such as nitrite (NO_2_^-^) or nitrate (NO_3_^-^), are food additives that must be included in the formulations of cured meat products. Although classified as antimicrobial preservatives, curing salts perform a variety of activities in the meat matrix, such as maintaining the colour and flavour (taste and aroma) properties of meat and acting as an antioxidant agent to ensure higher physicochemical stability (*1*, *2*). The use of these additives ensures that the sensory quality and stability of meat products are maintained throughout their shelf life, which is important for customer acceptance and satisfaction. Most cured products are ready-to-eat (RTE) and are not thermally treated before consumption. For this reason, ensuring the microbiological safety of these products is a major concern in the food industry. The use of additives, such as curing salts, is even more important in meat products with high pH (~6.5), as well as products with added mechanically separated meat, products stored at room temperature and products handled after processing or subjected to a variable cold chain, which is a particular concern in countries near the tropics, with high temperatures most of the year, and where food is transported over great distances (*3*, *4*).

The minimum amount of nitrite needed to assure good sensory properties and maintain microbiological safety is variable. In general, 50 to 75 mg/kg nitrite is needed to generate the characteristic cured colour, while a residual mass fraction of nitrite between 40 and 80 mg/kg is needed to effectively control microbes (*5*, *6*). As nitrites and nitrates are highly reactive, they react with different constituents of the meat and the added ingredients; these reactions are favoured by thermal processes, such as cooking. Therefore, the amount of nitrite or nitrate (hereafter referred to as nitrite/nitrate) added to the formulations is higher than the amount found in the final product and continues to decrease during storage (*1*). The amount of nitrite/nitrate that is permitted in the final product varies with the legislation of each country. In Brazil, the maximum residual mass fractions of nitrite (E249 and E250) and nitrate (E251 and E252) permitted in meat products are 300 and 150 mg/kg, respectively (*7*). In Europe, through Regulation (EU) 2023/2108 (*8*), the maximum nitrite allowed was reduced from 150 to 80 mg/kg in non-heat-treated cured meat products and from 100 to 50 mg/kg in heat-treated meat products. Furthermore, depending on the product, the maximum residual nitrite amount during the entire shelf life may not exceed 25-45 mg/kg. The Codex Committee on Food Additives (CCFA/FAO/WHO) has set a maximum limit of 80 mg/kg nitrite in meat products (*9*).

The nitrite and nitrates present in the human body can be exogenous in origin (when ingested through food, such as vegetables, water and meat products), or endogenous, such as nitrite and nitrates present in human saliva. According to Milkowski (*10*), 5 % of dietary nitrite and nitrate originates from meat products and the remainder originates from other foods such as vegetables. However, nitrites and nitrates differ significantly in their toxicity and permissible levels. The lethal dose of nitrite is approx. 300 mg/kg body mass, while that of nitrate is higher, at around 800 mg/kg body mass (*9*). The ingestion of nitrite may harm the human body and cause disorders, such as blood disorder-related methaemoglobinaemia, which results in a lack of oxygen in the cells (*4*). Nitrates, on the other hand, are generally considered less toxic because they first need to be converted into nitric oxide in the body (*4*). Despite these concerns, the consumption of nitrite and nitrate in meat products is not a concern due to the low available contents and the amount of meat products generally ingested. The potential health risks associated with the use of these additives mainly come from the generation of N-nitroso compounds (NOCs), especially N-nitrosamines. These compounds have mutagenic and genotoxic properties and thus have a strong oncogenic effect in humans, particularly with respect to colorectal cancer (*11*, *12*). NOCs can induce cancer in approx. 40 different animal species, including higher primates, and are carcinogenic in multiple organs in animals (*13*). Due to these concerns, the International Agency for Research on Cancer (IARC) concluded that ingested nitrates or nitrites are probable carcinogens to humans under conditions favouring endogenous nitrosation. Therefore, cured meat products were classified as a group 1 carcinogens (*11*).

N-nitrosamines are ubiquitous and are found in drinking water, animal or vegetable foods and drinks (*14*), in addition to cigarettes, pesticides and cosmetics. However, despite the ubiquity of N-nitrosamines in common environments, individuals argue that curing salts should be banned due to the potential presence of these compounds and the high contents of residual nitrite in meat products. Nevertheless, it is the responsibility of the meat industry to reduce or eliminate the risk of its products, as the scientific community is increasingly focused on designing better strategies to completely or partially replace conventional nitrites in meat products.

Although there is an undeniable need to reduce or remove curing salts from meat formulations, this task is challenging because curing salts have multiple purposes. In general, potential nitrite substitutes include the use of natural food dyes to obtain a ’cured colour’ similar to that of cured products (*15*, *16*), biopreservation techniques involving the use of bacteriocin, essential oils and nitrate-free plant extracts to guarantee microbiological safety (*17*-*19*), and the use of ’natural curing agents’, which replace nitrite with plant extracts that contain relatively high amounts of nitrate (*20*, *21*). The use of these techniques involves the partial reduction of nitrite or requires additional procedures to obtain all the effects (sensory, antimicrobial and antioxidant) conferred by curing salts (*22*). Replacing synthetic nitrites with ’natural’ nitrite sources is not better than using synthetic nitrite salts, but it is done to obtain a clean label on the product. Moreover, the labelling and advertising of these products as ’naturally cured’ can mislead the consumer, as the elimination of N-nitrosamines is not confirmed. The residual nitrite (and possible N-nitrosamine content) of ’naturally cured’ products is similar (or even higher) to that of products cured with synthetic nitrite or nitrate.

Recently, researchers have suggested the use of S-nitrosothiols (RSNO), which are derived from amino acids or peptides such as l-cysteine, glutathione and N-acetyl-l-cysteine, as nitric oxide (NO^•^) carriers for total substitution of curing salts in meat products (*23*-*26*). These carriers have been shown to have high potential, they can provide desirable sensorial and microbiological properties through mechanisms similar to those of nitrite, and can reduce the production of N-nitrosamines. However, research on the use of RSNO in meat products remains in early stages and there is very little information about its application at the industrial scale. Andrade *et al.* (*22*) reported that for nitrite mass fractions of 100–200 mg/kg, which are commonly used in cured cooked hams, equimolar amounts of RSNO (S-nitroso-N-acetylcysteine or S-nitroso-N-acetylcysteine ethyl ester) could achieve the same antioxidant effect, colour and volatile profile characteristics of these products, but produce lower amounts of residual nitrite.

The replacement of additives tends to depend on whether the characteristics of the food product can be maintained in some other way, which is challenging. Replacing nitrite/nitrate in the meat processing industry is complex because the food properties (composition, whole or reconstituted) and processes applied (cooking, fermentation, drying and smoking) are different. Thus, a proposed alternative to nitrite/nitrate should reduce the amount of N-nitrosamines while maintaining the sensory aspects of a product. Above all, the nitrite/nitrate alternative should be effective against foodborne bacteria, especially *C. botulinum*, which produces a lethal toxin. In this context, emerging nonthermal technologies have revolutionized the food processing sector, as they increase the shelf life of food and retain the quality, such as nutritional, freshness and sensory attributes, of food products (*27*). These technologies have great potential in reducing the health risks of cured meat products, either by allowing the reduction and/or even elimination of nitrite/nitrate salts.

## NITRITE/NITRATE EFFECTS

Although nitrate and nitrite salts are used as curing salts, the active curing agent is nitrite, which is produced by conversion of nitrate to nitrite with nitrate-reducing bacteria (species of *Achromobacter, Micrococcus, Lactobacillus* or *Staphylococcus*) that are naturally present in meat (*3*, *28*). Thus, nitrate salts are generally used as reserve agents for conversion to nitrite in products that require a long processing period.

**Fig. 1:**
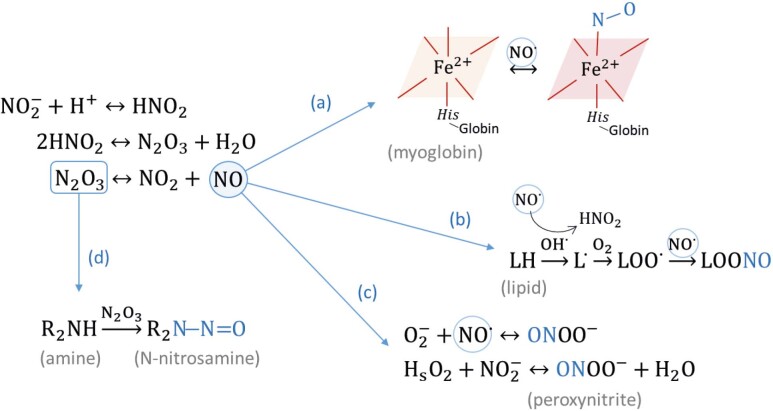
The mechanisms of nitrite actions in meat curing: (a) development of cured colour, (b) antioxidant effect, (c) antimicrobial effect, and (d) N-nitrosamine formation

Biochemically, most of the favourable actions of curing salts directly or indirectly result from the NO^•^ formed from the decomposition of nitrite in a solution. Likewise, the formation of undesirable compounds, such as N-nitrosamines, originates from the reactivity of intermediate components of these reactions (Fig. 1).

### Curing meat products

Soon after nitrite is added to the meat batter, the haem iron present in the deoxymyoglobin (DMb; MbFe^2+^) or oxymyoglobin (OMb; MbFe^2+^O_2_) is oxidized, as evidenced by the characteristic brown colour of metmyoglobin (MMb; MbFe^3+^). According to the chemical equilibrium of nitrosylated species, nitrite in an acidic medium (pH=5.8 to 6.2) produces nitrous acid (HNO_2_, pKa=3.42 at 25 °C), which is in equilibrium with its anhydrous dinitrogen trioxide (N_2_O_3_). Under reducing conditions and in the absence of light and oxygen, N_2_O_3_ decomposes into nitrogen dioxide (NO_2_^-^) and NO^•^. Then, NO^•^ reacts with DMb to form nitrosylmyoglobin (NOMb; MbFe^2+^NO) and/or with MMb to form nitrosylmetmyoglobin (MbFe^3+^NO), a transient pigment that is converted to NOMb (*1*, *29*). Thus, the binding of NO^•^ to myoglobin results in the pinkish-red colour pigment NOMb in the raw cured products or the pinkish colour nitrosyl-haemochrome (NOhemo) in the cooked cured products due to the denaturation of the pigment globin protein ((a) in Fig. 1). The formation of these pigments is responsible for the characteristic colours of these products (*30*).

In addition, NO^•^ stabilizes the haem iron of the meat pigments in cured products, limiting the pro-oxidant activity of iron. The presence of transition metals, especially iron (both haem and nonheme), is among the main factors that determine the oxidative stability of meat because they can catalyse different processes and stages of lipid oxidation (*31*). Lipid autoxidation is a complex process that involves the formation (initiation stage) and propagation of free radicals from lipids, beginning with the oxidation of unsaturated lipids by reactive oxygen species (ROS), such as hydrogen peroxide (H_2_O_2_), superoxide anion radical (O_2_˙) and hydroxyl radicals (OH˙). Then, lipid peroxyl radicals (L˙, LOO˙) and hydroperoxides (LOOH) are formed that are further degraded into secondary compounds (*2*, *29*). The main secondary compounds released include alcohols, ketones, alkanes, aldehydes, ethers and hydrocarbons, which are responsible for the deterioration of sensory aspects, such as odours and flavours associated with lipid oxidation (*31*). NO^•^ chelates free metals and sequesters oxygen and other ROS, inhibiting the initiation of lipid oxidation ((b) in Fig. 1). Moreover, NO^•^ shuts down lipid autoxidation by generating nonradical compounds and reacting with lipid peroxyl radicals (*2*). Therefore, the binding of NO^•^ to the haem iron of myoglobin prevents its oxidization and destroys the radical chain reactions of lipid oxidations, maintaining the characteristic flavour of cured meat products.

### Antimicrobial activity

Meat contains nutrients that, under ideal conditions, promote the growth of desirable and undesirable microorganisms. Desirable microorganisms are generally involved in the formation of sensory properties and stability of products, as occurs in the fermentation process. Undesirable microbes accelerate the deterioration of products and may cause disease in consumers. In general, the profile of the microbiota present in the meat product is influenced by the initial bacterial count in the raw material, the additional microbial load from the added ingredients, the sanitary conditions during handling, the processing techniques applied, the postprocessing handling method used, the type of packaging and the distribution and storage conditions (*32*).

Curing salts can extend the shelf life of meat and meat products by inhibiting the outgrowth of pathogenic and spoilage bacteria. The predominant bacteria associated with the spoilage of refrigerated meat products are *Brochothrix thermosphacta*, *Carnobacterium* spp, *Lactobacillus* spp, *Leuconostoc* spp and *Weissella* spp, which cause defects such as sour off-flavours, discolouration, production of gas or slime and decrease in pH. Nitrite has an inhibitory effect on the growth of several spoilage bacteria, such as *Enterobacteriaceae* and *B. thermosphacta*; however, the presence of nitrite combined with microaerophilic conditions favours the growth of psychotropic lactic acid bacteria (*33*). Nitrite is also recognized for its bacteriostatic and bactericidal effects against pathogenic bacteria such as *Escherichia coli*, *Salmonella* spp., *Listeria monocytogenes*, *Staphylococcus aureus* and *Clostridium* spp. (*2*, *3*).

The antimicrobial activity of nitrite is also attributed to reactions associated with the decomposition of nitrite to NO^•^ ((c) in Fig. 1). More precisely, this activity is directly related to the level of oxidative stress caused by peroxynitrite (ONOO^–^), which is formed by the reaction of nitrite with H_2_O_2_ and NO^•^ with O_2_‾˙ (*3*). Peroxynitrite is in equilibrium with peroxynitrous acid (ONOOH) under physiological conditions, and both compounds are powerful oxidants and nitrating agents. They lead to the oxidation and nitration of proteins, DNA and lipids through direct or indirect oxidative reactions and radical-mediated mechanisms (*2*, *3*). Therefore, the addition of nitrite improves the microbial safety of meat products.

Although different bacteria are affected by nitrite/nitrate, curing salts are added to control *C. botulinum* specifically, a strictly anaerobic Gram-positive rod-shaped (bacillus) bacterium that causes food poisoning (botulism) by a potent and lethal neurotoxin. Botulinic neurotoxins inhibit the release of acetylcholine at synapses and the electrical stimuli responsible for muscle contraction. These processes cause paralysis and suffocation, which can lead to death. It is estimated that poisoning can occur with the consumption of foods containing 30-50 ng of toxins (*34*). *C. botulinum* strains are classified as group I, II, III or IV according to the most prevalent way in which the bacterium obtains energy (proteolytic or saccharolytic mechanism) and the threshold and optimal temperature and pH for growth. With respect to public health, the most dangerous spore-formers in low-acid chilled foods (such as meat) are from group II, the psychrotrophic nonproteolytic strains, which can germinate and grow at temperatures as low as 3 °C (*35*). Although lower storage temperatures (less than 2 °C) tend to reduce or impede the production of toxins, maintaining this temperature throughout the distribution and storage chain to the point of sale is very challenging, especially in developing countries. Therefore, nonproteolytic *Clostridium* can only be controlled through the presence of residual nitrite, which prevents the germination of spores and, consequently, inhibits the production of toxins.

Nitrites show antibotulinal activity in a heated system by preventing vegetative cell growth from spores and inhibiting the cell division of any vegetative cell. In addition to the action of the peroxynitrite formed, the main inhibition of *C. botulinum* by nitrite may involve a reaction between ^•^NO and important iron-sulphur enzymes (*e.g.* ferredoxin and pyruvate-ferredoxin oxidoreductase) necessary for energy production in clostridial vegetative cells (*36*, *37*). However, despite the high lethality of *C. botulinum*, the survival or growth of this microorganism in the reformulated product is not considered in many studies on the replacement and/or reduction of nitrite in meat products.

### N-nitrosamine formation

N-nitrosamines are a class of compounds that contain a nitrous group (-N-N=O) attached to a secondary amine (R_2_-), which can react with different radicals (generally hydrocarbons), forming a wide variety of compounds. Once present in the human body, these compounds are metabolized by enzymes and produce the diazonium radical, which is a very reactive electrophile that reacts with cellular macromolecules, such as nitrogenous bases (nucleophilic species) present in DNA (*12*).

N-nitrosamines are divided into volatile N-nitrosamines and non-volatile N-nitrosamines. The former are typically potent carcinogenic nitrosamines present in processed meat products, including N-nitrosodimethylamine (NDMA), N-nitrosodiethylamine (NDEA), N-nitrosopiperidine (NPIP), N-nitrosopyrrolidine (NPYR), N-nitrosomorpholine (NMOR), N-nitrosomethylethylamine (NMEA), N-nitroso-di-n-propylamine (NDPA) and N-nitrosodibutylamine (NDBA). Among the volatile N-nitrosamine compounds which have been studied, only 18 % are considered noncarcinogenic, while the non-volatile N-nitrosamines are weakly carcinogenic or assumed to be noncarcinogenic (*38*). The lack of carcinogenic potential by some N-nitrosamines is attributed to their chemical structure. Species with high-molecular-mass radicals and long carbon chains are less susceptible to metabolization and consequently the release of diazonium radicals due to steric hindrance. Therefore, N-nitrosamines of lower molecular mass, such as NDMA and NDEA, have the highest mutagenic potential (*12*).

In meat, N-nitrosamines are formed during processing through reactions between secondary amines and nitrosating agents, such as N_2_O_3_, which are derived from the curing reactions of nitrite to ^•^NO ((d) in Fig. 1) (*12*, *38*). In the food processing industry, ascorbate or iso-ascorbate (or erythorbate) salts are commonly used to accelerate curing reactions and, consequently, reduce processing time and the availability of nitrosating agents to form N-nitrosamines (*3*). However, according to Majou and Christieans (*3*), ascorbate can also participate in the reduction cycle of metmyoglobin reductase (MMR) and generate ˙NO from nitrite, which binds to DMb and maintains the production of O_2_˙ and H_2_O_2_. These reactions increase the production of peroxynitrite and promote antimicrobial activity.

The N-nitrosamines found in cured meat products are called preformed N-nitrosamines and their inhibition during processing remains a major challenge for the safety of meat products. The mass fractions of N-nitrosamines in meat products vary widely, from undetectable (<1 μg/kg) to several thousand µg/kg, depending on the type of N-nitrosamines (*14*, *38*). In general, exposure of the product to high temperatures during processing or preparation for consumption tends to increase the content of pre-formed nitrosamines. This occurs because higher temperatures catalyse the formation of essential substrates for the N-nitrosation reaction, such as nitrogen oxides and secondary amines (*12*). However, in addition to the N-nitrosamines found in different types of food products (*14*), they can also be formed endogenously during food digestion under acidic conditions (pH close to 3.0) in the human stomach. It is estimated that endogenous formation contributes 45–75 % of the total human exposure to NOC (*39*). According to Hinuma *et al.* (*40*), the stomach is an important place for nitrosation because the precursors of N-nitrosamines, such as secondary amines and nitrite, enter the stomach in foods with saliva and the acidity of gastric juice is a suitable medium for nitrosation. Importantly, nitrite is also formed in the human body from the reduction of nitrate by bacteria in the oral cavity; therefore, humans are primarily exposed to nitrite from the consumption of vegetables and water, which are the main sources of nitrate intake (*4*, *40*).

## NONTHERMAL TECHNOLOGIES

Antimicrobial technologies in food processing can be classified as thermal or nonthermal. During thermal processing, food is preserved by exposure to a very high temperature, which decreases microbial growth or contamination from food but also leads to some deleterious effects on food products, such as a loss of heat-sensitive nutritional components, textural changes, changes in rheological properties and changes in sensory attributes (*27*). In nonthermal processing, food is processed at near room temperature and heat-sensitive nutritious compounds are not damaged. These processes cause a reduction in the microbial load in food and thus increase shelf life, and also achieve good sensory and textural characteristics (*41*). Different researchers have proposed the use of nonthermal techniques to reformulate meat products by reducing or replacing curing salts and improve the quality of products and meet consumer expectations.

### Irradiation

Food irradiation involves exposing food to a controlled amount of ionizing radiation (energy). With irradiation, little or no heating is applied to the food matrix, nothing is added to the food and less energy and water are needed for processing. Additionally, irradiation can be applied to either packaged or bulk food in large or small quantities. The amount of energy absorbed, which is expressed in gray (Gy) – equivalent to one unit of joules per kilogram of food (J/kg), can be disrupted by several factors, including physicochemical properties (temperature, physical state and density) of the food to be irradiated, the dose applied, the exposure time and the distance of the food to the radiation source (*42*, *43*).

Ionizing radiation can be classified into high-speed electrons, alpha (α, composed of two protons and two neutrons) and beta (β, composed only of electrons), and elementary particles of electromagnetic waves, X-rays and gamma rays (γ), which have zero charge and higher energy (*43*). Three types of radiation used to treat foods show different penetration and effects with respect to the microbiological and physicochemical quality of foods (*44*). Gamma particles can penetrate matter more intensely by emitting continuous rays at a predictable rate and, therefore, are the most important type of radiation in the food processing industry. In general, the radioactive isotopes cobalt-60 (^60^Co) and caesium-137 (^137^Cs) are used for the emission of gamma rays (*41*, *43*). However, the future use of these isotopes is uncertain due to the rising prices of cobalt-60, increased public concerns about the safety of radioactive materials and issues related to their safe disposal (*44*). This safety concern has provided an opportunity for the application of electron beams in food products; electron beam accelerators are used to generate high-energy electrons (of 10 MeV) that can penetrate up to 39 mm deep in food with high moisture content (*41*). As the energy of ionizing radiation is high but lower than the threshold of nuclear reactions, food does not become radioactive (*43*). Therefore, the use of ionizing radiation in food processing has been approved and regulated in as many as 60 countries.

Irradiation can increase the shelf life of foods and their safety. The process promotes chemical, physical and biological changes in the structure of microorganisms, preventing their multiplication. Direct irradiation leads to the unfolding of DNA and damage to nucleic acids, and the ionization of water molecules results in oxidative damage to microbial cells (*41*, *42*). According to Ehlermann (*45*), the effects of the application of irradiation in food processing to control the growth of microorganisms can be classified as radappertization, radicidation or radurization, which are differentiated by the dose of radiation applied. Radappertization involves the use of extremely high doses of radiation ranging from 30 to 50 kGy. These doses can kill all microorganisms in foodstuffs, including spores, and are typically used for sterilization. During radicidation, moderate doses of radiation (between 1 and 10 kGy) are used to reduce spoilage and microbial pathogens, resulting in food pasteurization. Low doses (less than 1 kGy) are used in the radurization process to inhibit respiration, control microbial growth in fresh meat, slow the ripening of vegetables and prevent infestations of grains and fruits with insects and parasites.

Irradiation alters food components in meat, particularly lipids and proteins, but can preserve the original quality of the meat (*46*). Fats begin to oxidize when exposed to radiation, leading to rancid off-flavours, oxidation of the meat pigment and unfavourable colour changes (*47*, *48*). Nevertheless, food irradiation at doses up to 10 kGy does not cause any nutritional issues (*46*).

Recent results of the applied irradiation during cured meat processing related to microbial control and/or N-nitrosamine formation are shownin Table 1 (*5*, *49*-*55*). When doses lower than 6.0 kGy were applied, a significant, dose-dependent reduction in vegetative cells of spoilage microorganisms and pathogens was reported (*49*, *50*). In cooked ham, Silva *et al.* (*51*) reported that the number of recovered *C. sporogenes* spores decreased with increasing doses of radiation (up to 6.0 kGy), but the radiation sensitivity (D-values) decreased from 1.98 to 1.81 kGy in uncured samples and to 1.57 kGy in cured samples. According to these authors, the microbiological safety was guaranteed with 50 mg/kg nitrite and irradiation at a dose of 1.5 kGy. Nevertheless, Dutra *et al.* (*5*) reported that gamma irradiation higher than 10 kGy could inactivate *C. botulinum* spores, regardless of the addition of nitrite salts. However, the resistance of spores to irradiation depends on multiple factors, such as the type of spores (proteolytic or nonproteolytic strains), the temperature of the product and the environment, the treatment medium and the initial count of microorganisms. Proteolytic *C. botulinum* spores are more resistant to irradiation than nonproteolytic strains (*56*).

**Table 1:** Recent research on the application of gamma irradiation for microbial control and reduction of N-nitrosamines in meat products

Reference	Product	Assessment	Main conclusions
Dutra *et al.* (*5*)	Mortadella	Effect of different gamma irradiation doses (0, 10, and 20 kGy) and the addition of *w*(nitrite)=0, 150 and 300 mg/kg on products inoculated with 10^7^ *Clostridium botulinum* spores/g.	Gamma irradiation of 10 kGy is capable of inactivating *C. botulinum*, regardless of the addition of nitrite salts.
Silva *et al.* (*49*)	Cooked and sliced ham	Effect of different gamma irradiation doses (0.5, 1.0, 1.5 and 2.0 kGy) and the addition of *w*(nitrite)=0, 50 and 150 mg/kg on products inoculated with 10^6^ CFU/g *Listeria monocytogenes*.	There was a synergistic effect between nitrite and irradiation treatments in eliminating *Listeria*, namely *w*(nitrite)=150 mg/kg combined with 1.5 kGy, and *w*(nitrite)=50 mg/kg with 1.8 kGy.
Rodrigues *et al.* (*50*)	Sausage	Effect of different gamma irradiation doses (1.5, 3.0, and 4.5 kGy) and *w*(salt)=2 and 1.25 % on microbiological safety and shelf life of cured (*w*(nitrite)=150 mg/kg) cooked sausages.	The reduction in microbial load was dose dependent; the minimum dose (1.5 kGy) resulted in reductions of up to 6 log cycles of lactic acid bacteria.
Silva *et al.* (*51*)	Cooked ham	Effect of different gamma irradiation doses (0, 1.5, 3.0, 4.5 and 6.0 kGy) and *w*(nitrite)=0, 50 and 150 mg/kg on products inoculated with 10^7^ *Clostridium sporogenes* spores/g.	The microbiological safety was guaranteed with *w*(nitrite)=50 mg/kg and 1.5 kGy irradiation.
Wei *et al.* (*52*)	Chinese Rugao ham	Effect of gamma irradiation (5 kGy, rate 10-15 kGy/h) on the profile of biogenic amines, N-nitrosamines and residual nitrite in products with added *w*(nitrite)=150 mg/kg.	Irradiation was able to reduce the residual nitrite content. The amounts of some biogenic amines (tyramine and putrescine) decreased and others (spermidine, cadaverine, tryptamine and phenylethylamine) increased. Irradiation is capable of degrading volatile N-nitrosamines.
Ahn *et al.* (*53*)	Smoked sausage	Effect of different gamma irradiation doses (0, 5, 10 and 20 kGy) on products with added *w*(nitrite)=150 mg/kg.	The application of 10 and 20 kGy reduced the formation of N-nitrosodimethylamine (NDMA) in the vacuum-packed product or under aerobic conditions. N-nitrosopyrrolidine (NPYR) was not detected in irradiated samples, regardless of concentration.
Ahn *et al.* (*54*)	Sausage	Effect of applying different irradiation doses (0, 5,10, 20 and 30 kGy) on products with added *w*(nitrite)=156 mg/kg, stored in aerobic and anaerobic packaging.	Irradiation to control nitrosamines was more effective in products stored in aerobic packaging, with a reduction effect starting at 10 kGy. In anaerobic packaging, a minimum dose of 20 kGy was required.
Ahn *et al.* (*55*)	Sausage	Effect of gamma irradiation (0, 5, 10 and 20 kGy) and the addition of *w*(nitrite)=75 and 150 mg/kg on ​​N-nitrosamine content during storage for 4 weeks at 4 °C.	The effect of irradiation on N-nitrosamines was observed with storage. A dose equal to or greater than 10 kGy was necessary to control N-nitrosodimethylamine (NDMA) and N-nitrosopyrrolidine (NPYR).

CFU=colony forming units

The radiolysis (dissociation of molecules) caused by gamma irradiation also helps reduce the mass fraction of residual nitrite and N-nitrosamines in meat products. Furthermore, in irradiated products nitrites cannot function as nitrosating agents. Therefore, the formation of N-nitrosamines can be inhibited (*53*-*55*, *57*, *58*). Although positive bactericidal effects and N-nitrosamine degradation have been verified, the use of irradiation may be limited due to changes in the sensory properties of the product. Considering that radiolysis is not selective, the lysis of other substances, such as water, can also occur. This process releases hydroxyl radicals (OH˙), which can increase the extent of lipid oxidation in food products. Lipid oxidation induced by radiolysis produces compounds such as sulfurates that can negatively change aroma and taste, impairing the sensory acceptance of meat products (*50*, *59*-*61*). Although irradiation intensifies lipid oxidation, Dutra *et al*. (*60*) observed that adding nitrite to emulsified sausages had a greater effect than irradiation on the quality parameters evaluated. Even at low mass fractions (∼75 mg/kg), the use of irradiation decreased the deleterious effects of irradiation at doses as high as 20 kGy.

Therefore, the use of irradiation has great potential as a tool to reformulate meat products aimed at reducing nitrite amounts, contributing to the reduction of residual nitrite and N-nitrosamines while ensuring microbiological safety. However, the amount of nitrite reduction and irradiation dosage applied must be evaluated for each specific meat product (emulsified, fermented, smoked, *etc*.), as well as the technical and financial feasibility of this type of application. Moreover, it is crucial to change the current consumer perceptions about irradiation since it restricts the growth of this technology in the food industry sector.

### Plasma

Cold plasma is an emerging, economical and environmentally friendly technology with potential applications in the food and bioprocessing industry, including microbial decontamination, enzyme inactivation, shelf-life extension and physicochemical modification (*62*).

Plasma is the fourth state of matter and is formed by gases subjected to extremely high energies. As a result, molecular agitation overcomes the energy of external electrons. Then, the released electrons form a shapeless, electrically neutral mass composed of electrons and dissociated nuclei (*63*). Basically, plasma treatment can be divided into two types: thermal plasma and cold plasma (nonthermal). Thermal plasma produces a large amount of energy by using high temperature. Cold plasma is a nonthermal treatment that works in the temperature range of 25–65 °C (*41*, *64*). It can be induced and sustained through an electric discharge in a gas at atmospheric or low pressures, using the corona, dielectric barrier discharge (DBD) or gliding arc discharge configurations (*65*). Cold plasma can be obtained using air and feed gases, such as helium, nitrogen and argon, or a mixture of gases, which are subjected to different energy levels to increase the kinetic energy of electrons. Depending on the discharge gas, the cold plasma process yields different biologically active agents: reactive oxygen species (ROS), including atomic oxygen, ozone (O_3_), hydroxyl radical (OH˙) and hydrogen peroxide (H_2_O_2_); and reactive nitrogen species (RNS), such as peroxynitrite (ONOO˙), nitrate (NO_3_‾), nitrite (NO_2_‾), their corresponding acids, and nitrogen oxides (NO_x_) (*63*, *64*). The reactive species and their concentration in the plasma vary depending on many factors, including the gas in which plasma is induced, the configuration of the plasma source, the power input to the gas, the duration of treatment and the humidity levels (*65*).

In food processing, cold plasma is an effective strategy for controlling microbial development. The ROS generated from plasma detrimentally interact with vital cellular biomolecules, such as DNA, proteins and enzymes, in cells and may alter the function of biological membranes *via* interactions with lipids. This process causes unsaturated fatty acid peroxides to form and amino acids in proteins to oxidize (*56*, *64*, *65*). The mechanism of action of cold plasma is different for Gram-positive and Gram-negative bacteria. Cleavage of specific peptidoglycan bonds and oxidation of lipids present in lipopolysaccharides occur in Gram-negative bacteria, while oxidative damage to intracellular components, such as DNA, occurs in Gram-positive bacteria (*66*). The effectiveness of the antibacterial action depends on numerous factors, including the type of plasma discharge, operating conditions, choice of gas, relative humidity of the product, state (solid or liquid) of the food and the microorganisms present (*65*).

Due to the formation of reactive nitrogen species (RNS) during its application, plasma can generate a ’natural’ source of nitrite. Therefore, in meat processing, cold plasma has been used to induce the curing process and is applied postprocessing (*67*). In addition, cold plasma can be applied directly into the meat batter during the processing steps (*68*) or indirectly when applied to water (*63*, *69*-*71*) or some ingredient (*72*, *73*) of the formulation. When plasma is applied to water that is added to the formulation, this mixture can be considered an additive. According to Jung *et al.* (*69*), the application of plasma in deionized water can favour its acidification (pH decreases from 7 to 2), and a concentration of 782 mg/L nitrite and 358 mg/L nitrate can be formed after 120 min. When the meat mass is directly exposed to cold plasma, a mass fraction of 66 mg/kg nitrite is formed after 30 min of application (*68*). In general, the amount of nitrite production tends to increase with increasing plasma potency (*70*), but since the application of cold plasma increases the temperature of the meat mass, the optimal nitrite mass fraction without exceeding the recommended temperature (<13 °C) was reported to be 42 mg/kg (*71*). Nevertheless, as previously described, it is important to highlight that replacing synthetic nitrites with ’natural’ nitrite sources does not provide healthier or safer advantages over synthetic nitrite salts.

Similar to the irradiation process, cold plasma produces free radicals and reactive species *via* gaseous electrical discharge that act as antibacterial agents but can also induce lipid oxidation, especially during the storage of meat products (*62*, *72*). Lipid oxidation induced by an oxygen-containing cold plasma process can affect the acceptability and shelf life of foods. The nutritional, sensory and other quality attributes can change depending on the cold plasma process, time and exposure conditions. In the case of solid foods, however, the lower penetration depth of plasma and the reactive species (10–50 µm depth) affect only the surface and help retain nutrients inside the product (*64*).

In Table 2 (*63*, *72*-*75*), we summarize the results obtained to date from cold plasma treatment of meat, both in terms of microbial control (other than *C. botulinum*) and minimization of N-nitrosamines. Although the application of cold plasma in meat processing has high potential, this decontamination technology is a relatively newer approach in the food industry and more research is needed to optimize it for specific applications in meat processing. Since there are no reports on the effect of cold plasma treatment on *C. botulinum* growth and N-nitrosamine mass fractions, further research is needed to clarify its effectiveness in reducing the risks associated with the use of nitrite/nitrate in meat products.

**Table 2:** Recent research on the application of cold plasma for microbial control and reduction of N-nitrosamines in meat products

Reference	Product	Assessment	Main conclusions
Inguglia *et al.* (*63*)	Beef jerky	Effect of applying cold plasma (20 kHz, supplied with air or nitrogen) in brine water with *w*(nitrite)=0, 50 and 150 mg/kg in products inoculated with *Listeria innocua*.	Only plasma-treated samples had residual nitrite mass fractions. A reduction of 0.5 log CFU/mL of *Listeria* was verified in the samples treated with plasma, regardless of the nitrite content.
Marcinkowska-Lesiak *et al.* (*72*)	Sausage	Effect of plasma application in plant solutions (soybean and lentil) as a substitute for *w*(nitrite)= 75 mg/kg on microbiological and technological characteristics.	There was no difference in residual *w*(nitrite)=50 mg/kg and in the development of aerobic bacteria, regardless of the treatment.
Kim *et al.* (*73*)	Sausage	The effect of adding onion powder treated with plasma (550 W and 25 kHz) and without sodium nitrite on carcinogenic potential was evaluated using the Ames test.	Onion powder treated with plasma did not show an increase in the number of His+ revertant colonies, suggesting safety for application.
Yong *et al.* (*74*)	Beef jerky	Effect of applying cold plasma (4 kHz) for 0, 20, 40 and 60 min after marinating pork meat in brine (with and without nitrite).	The application of cold plasma for 40 min was sufficient to reach the same nitrite mass fraction as in the conventional formulation (*w*(nitrite)=150 mg/kg). Higher antibacterial action against *Staphylococcus* spp. and *Bacillus* spp. was verified in samples treated with cold plasma.
Gök *et al.* (*75*)	Pastrami	Effect of application time (0, 180 and 300 s) and composition of oxygen, argon and two oxygen/argon mixtures (25:75 and 50:50) of cold plasma on the control of *Staphylococcus aureus* (10^6^ CFU/g) and *Listeria monocytogenes* (10^6^ CFU/g).	The counts of *S. aureus* (0.85 log CFU/cm^2^) and *L. monocytogenes* (0.83 log CFU/cm^2^) were reduced, regardless of the time of application. Plasma from oxygen gas was more effective for the reduction of *L. monocytogenes*.

CFU=colony forming units

### High-pressure process

High hydrostatic pressure (HHP) technology is applied in food processing mainly to control microbes through enzyme inactivation (*27*, *76*). In an HPP system, the high pressure (100–1000 MPa) is uniformly distributed by an incompressible (hydraulic) fluid, usually water, to packaged food in a thermally insulated airtight vessel. This process enables a pasteurization effect through the uniform and instantaneous application of isostatic pressure for approx. 10–20 min. According to the isostatic principle, when pressure is applied to a liquid medium in a closed environment, equal pressure is applied to the food product at any point, regardless of its size and shape. Therefore, food products of different volumes can be processed within the same batch. Moreover, HPP reduces the influence of temperature on food components. Although the effect of high pressure induces adiabatic heating in water at 3 °C every 100 MPa, the initial pasteurization temperature is controlled within 5–10 °C so that the water temperature will not exceed 30 °C when the pressure reaches 600 MPa (*77*, *78*). The efficiency of the application of HPP can be affected by several parameters related to the process (such as adiabatic heating, decompression time, pressure strength, combination of time and pressure, and packaging material), as well as factors intrinsic to the product (pH, water activity, composition, *etc*.) and extrinsic factors (storage and transport conditions) (*27*).

In addition to increasing the health benefits of food products, HPP is a nonthermal technique that potentially enhances the shelf life of food and maintains its quality and safety. In addition to inactivating pathogens, this technology provides several positive effects, as it better preserves the flavour compounds and provides better nutritional value than conventional thermal methods (*41*). HPP acts in the range from 5 to 30 °C, which reduces the occurrence of the Maillard reaction and caramelization and prevents the release of new aromatic compounds. Consequently, the use of colouring and flavouring agents for meat products is reduced. On the other hand, HPP can affect the microstructure and negatively affect the texture and water retention capacity of certain meat products (*79*). Regarding food safety, although high pressure can eliminate pathogens, a large variety of microbes with different physiological characteristics occur in food products, which leads to highly diverse pressure tolerance characteristics among these microbes. HHP treatment is more effective against eukaryotes, Gram-negative bacteria, protozoa and parasites than against yeast and mould, which inactivate at much higher pressures (*27*, *77*).

In recent years, the success of HHP against important pathogens in meat products has been reported in several studies (Table 3 (*80*-*89*)). HPP has antibacterial activity by modifying the morphology of the microorganism, which causes damage in the cell membranes, the denaturation of proteins and enzymes involved in important genetic mechanisms and chemical reactions that inhibit the growth of microorganisms. The inactivation of microorganisms by HPP may also be related to the rupture of the cell membrane, the formation of ripples and subsequent swelling and reduction in cell volume (*78*). However, although HPP inactivates bacteria, it only partially inactivates spores. Moreover, *C. botulinum* spores are very resistant to HPP, especially at room temperature. In minced raw chicken meat inoculated with five nonproteolytic strains of *C. botulinum*, which were cooked and treated at 600 MPa for 2 min at 20 °C, Linton *et al.* (*80*) observed that the spores survived after treatment and germinated during storage. Factors related to bacteria (type, initial count, *etc*.), food matrix (pH, water activity, *etc*.) and treatment conditions (pressure cycles, temperature, *etc*.) affect the resistance of cells and spores to the applied HPP or even the combination of treatments. Similarly, spore recovery after HPP application depends on the medium, additives and incubation conditions (*56*). Therefore, many researchers have combined HHP with sodium nitrite or other antibacterial agents (bacteriocin, plant extract, essential oil, *etc*.) to reduce the addition of sodium nitrite or produce a synergistic antibacterial effect.

**Table 3:** Recent research on the application of high hydrostatic pressure (HHP) for microbial control and reduction of N-nitrosamines in meat products

Reference	Product	Assessment	Main conclusions
Linton *et al.* (*80*)	Cooked chicken	Effect of HHP applied at 600 MPa and 20 °C for 2 min on uncured cooked minced chicken without nitrite and inoculated with five nonproteolytic strains of *C. botulinum* (10^4^ spores/g). They investigated oxygen-permeable packaging, and the addition of 2 % sodium lactate and *Weissella viridescens* (10^8^ CFU/g) to control *C. botulinum*.	The HPP treatment does not inactivate bacterial endospores and the conditions in cooked chicken support the growth of *C. botulinum*. *W. viridescens* was not suitable for use as a protective culture to control the growth of *C. botulinum,* but germination and growth were controlled with the addition of 2 % sodium lactate and the use of oxygen-permeable packaging.
Higuero *et al.* (*81*)	Dry-cured sirloin (sliced)	Effect of HHP treatment at 600 MPa for 7 min on technological and physicochemical properties of products made with reduced amounts of sodium nitrite and nitrate (*w*=150, 75, 37.5 and 0 mg/kg), sliced and stored under refrigeration for 240 days.	The HPP treatment applied (600 MPa, 7 min) minimally affected the colour and oxidation of lipids and proteins, regardless of the added sodium nitrite/nitrate amount. The reduction of nitrate/nitrite up to 75 % proved to be feasible without affecting the colour, lipid and protein oxidation.
Lopez *et al.* (*82*)	Fermented sausage	Effect of HHP applied at 593 MPa for 290 s on fermented sausages made without nitrite and inoculated with *Listeria innocua* (10^6^ CFU/g) and *Salmonella enterica* (10^6^ CFU/g) strains.	The count of *L. innocua* and *Salmonella* spp. was reduced by up to 4.74 and 3.83 log CFU/g, respectively, in sausages treated with HHP.
Balamurugan *et al.* (*83*)	Cooked sausages	Effects of HHP (600 MPa for 3 min) and hot water (75 °C for 15 min) pasteurization on the inactivation of inoculated *Listeria monocytogenes* (10^8^ CFU/g) in vacuum-packaged cooked sausages and their recovery during storage at 4 and 10 °C for 35 days.	The combined effect of HPP and hot water resulted in a >6 log reduction in the number of *L. monocytogenes* inoculated during storage, regardless of the temperature.
Lavieri *et al.* (*84*)	Restructured ham (sliced)	To investigate the effect of 0.50 and 100 mg/kg of vegetable source nitrite and the use of HHP at 400 and 600 MPa for 4 min on the *Listeria monocytogenes* count (10^5^ CFU/g) inoculated into slices of restructured ham.	Treatment with HHP of 600 MPa for 4 min led to populations of *L. monocytogenes* below the limit of detection, independent of the added nitrite mass fraction.
Myers *et al.* (*85*)	Cooked ham (sliced)	Effect of added nitrite source (chemical or vegetable powder) and HHP (600 or 400 MPa for 3 min) on *Listeria monocytogenes* (10^6^ CFU/g) growth and physicochemical and technological characteristics.	The application of HHP at 600 MPa, regardless of the nitrite source used, reduced the number of *L. monocytogenes* by more than 3 log CFU/g, while the use of 400 MPa had a minimal effect. Furthermore, the use of HHP did not change the mass fraction of residual nitrite in the formulations.
Myers *et al.* (*86*)	Cooked ham and turkey (sliced)	Effect of HHP treatment at 600 MPa for 3 min on products made with different meat species and *w*(sodium nitrite)=0 and 200 mg/kg, both inoculated with 10^5^ CFU/g *Listeria monocytogenes*, during 28 days of refrigerated storage.	The use of HHP reduced *L. monocytogenes* counts by more than 3 log CFU/g after 182 days of storage.
Cava *et al.* (*87*)	Salchichón and dry-cured loin	The effect of applying HPP (600 MPa, 8 min) on the control of *L. monocytogenes* was evaluated in products made with w(nitrite)=150 mg/kg, sliced ​​and vacuum-packed.	Average reductions of 2.30 log CFU/g in the *L. monocytogenes* population were observed immediately after high-pressure treatment.
Pietrzak *et al.* (*88*)	Cooked ham	Effects of HHP (600 MPa, 10 min, 20 °C) on the control of *Salmonella*, coliforms and *Staphylococcus aureus* in nitrite-reduced ham (50 and 80 mg/kg).	The reduction of sodium nitrite did not affect the microbiological quality after 8 weeks of storage when treated with high pressure.
Hayman *et al.* (*89*)	Pastrami, Strassburg, sausage and Cajun meat	Effect of HHP (600 MPa, 20 °C, for 180 s) on shelf life extension and safety against *L. monocytogenes*.	High pressure was efficient in controlling aerobic and anaerobic bacteria, generating counts below detectable limits and lower than untreated ones after 95 days of storage at 4 °C.

CFU=colony forming units

The application of HHP on an industrial scale has been recommended, mainly for postprocessing and ready-to-eat (RTE) packaged products. In this sense, the use of HHP as a sterilization treatment helps reduce the need to add nitrite to meat products, meeting the growing consumer demand for natural, preservative-free and minimally processed products. However, research on this technology is scarce, especially regarding the effects on the survival of *C. botulinum* and the quantification and formation of N-nitrosamines in cured meat products.

### Prospects

Some additional nonthermal technologies, such as pulsed electric field (PEF) and ultrasound, have not been tested as strategies for replacing nitrite in meat products. However, promising results have been obtained in the reduction of other ingredients, such as salt (sodium chloride) and phosphate, with increased diffusion of salting (*90*-*92*).

PEF processing involves repeated applications of pulses with an electric field strength of 0.1–100 kV/cm to the food between the electrodes. The resulting electric field causes the formation of pores in the cell membranes of microbes (*93*). Although the application of PEF in solid foods is less studied than its application in liquid foods (*41*), in which researchers aim to sterilize and inactivate enzymes, this technology may reduce the amount of sodium nitrite and the curing time needed by disrupting cellular tissues and affecting the mass transfer processes (*90*).

Ultrasonication is used at different frequencies, including low (20–100 kHz), medium (100 kHz−1 MHz) and high frequency (1–100 MHz). Low-frequency ultrasonication causes strong shear forces in the medium, while high-frequency ultrasonication causes weaker shear force in the medium (*41*). The propagation of mechanical energy by ultrasonic waves results in a captivation effect, which is characterized by compression (negative pressure) and decompression (positive pressure) zones. The resulting gas bubbles can implode when unstable, promoting an increase in temperature and pressure that exerts mechanical, chemical and biochemical effects (*94*). Due to the effects caused by cavitation and other acoustic phenomena, ultrasound can affect the technological properties of proteins and meat products. Thus, this technology has already been applied to reduce sodium chloride and additives such as phosphate (*91*, *92*).

## CONCLUSIONS

In addition to ensuring the sensory quality and food safety of meat products, the use of curing salts can promote the formation of N-nitrosamines. Completely replacing curing salts is challenging due to the versatility and effectiveness of these additives in developing the sensory properties of food products and controlling microbial growth. Thus, a validated alternative to curing salts that can be applied on an industrial scale is lacking in the food processing industry. Most alternative technologies have not adequately reduced the use of N-nitrosamines in meat products. Combining technologies may be an effective alternative to curing salts, limiting the generation of N-nitrosamines without impairing the sensory properties of the product.

However, there are gaps in the relevant research on antimicrobial effects, particularly the effects of these alternative techniques on highly toxic *Clostridium botulinum*. The difficulty in performing tests on highly resistant spores of *C. botulinum* is among the main challenges in this field of food safety research. *C. botulinum* spores are generally resistant to high hydrostatic pressure and irradiation, but promising results in terms of inactivation have been achieved when one of these techniques was combined with heat treatment. Likewise, nonthermal technologies can be used to exert synergistic effects on the inactivation of *C. botulinum*.

Although nonthermal technologies generally require higher initial investments, maintenance and high energy inputs than conventional thermal processes, these methods achieve high operational efficiency. Future research related to nonthermal technologies should focus primarily on increasing the cost-effectiveness and viability of these technologies to broaden their production and marketing scope in the food processing sector. Through this research, nutritious and safe food can be produced without eliminating the sensory properties that make these foods palatable to consumers.
